# The seismic wavefield as seen by distributed acoustic sensing arrays: local, regional and teleseismic sources

**DOI:** 10.1098/rspa.2021.0812

**Published:** 2022-02

**Authors:** B. L. N. Kennett

**Affiliations:** Research School of Earth Sciences, The Australian National University, Canberra, Australian Capital Territory 2601, Australia

**Keywords:** distributed acoustic sensing, array response, strain rate

## Abstract

Distributed acoustic sensing (DAS) exploiting fibre optic cables provides high-density sampling of the seismic wavefield. Scattered returns from multiple laser pulses provide local averages of strain rate over a finite gauge length. The nature of the signal depends on the orientation of the cable with respect to the passing seismic waves. For local events, the dominant part of the strain rate can be extracted from the difference of ground velocity resolved along the fibre at the ends of the gauge interval. For more distant events the response at seismic frequencies can be represented as the acceleration along the fibre modulated by the wave slowness resolved in the same direction, which means there is a strong dependence on cable orientation. Slowness–frequency representations of the wavefield provide insight, via modelling, into the character of the DAS wavefield in a range of situations from a local jump source, through a regional earthquake to teleseismic recording. The slowness-domain representation of the DAS signal allows analysis of the array response of cable configurations indicating a bias due to the slowness weighting associated with the effect of gauge length. Unlike seismometer arrays the response is not described by a single generic stacking function.

## 1. Introduction

Local distortions are produced as a dynamic disturbance interacts with an optical fibre. The distortions affect the way that light propagates along the fibre. Minor imperfections in the fibre act as scatterers, so that variations in the properties of backscattered light can be used to map out the passage of waves past the fibre.

With repetitive sampling by laser pulses, interferometric sensing can be used to measure the phase of the backscattered light as a function of time from the emission of the pulse, and hence the position along the fibre. The relative phase along the fibre provides a measure of local strain or strain rate, and so a single fibre can become a large suite of effective sensors (e.g. [1,2]). The use of such multiple measurements of strain or strain rate extracted from Rayleigh backscattering has been termed distributed acoustic sensing (DAS). Although DAS results are presented as time series at specific points, the nature of the sampling process means that there is averaging over a ‘gauge length’ around the nominal point.

The dense sampling capabilities of DAS have seen a wide range of geophysical uses. The initial developments came in exploration geophysics with borehole installations (e.g. [3–5]), and the dense sampling offers considerable potential for surface profiling (e.g. [6]). There have been a wide range of DAS applications in seismology from local monitoring to the analysis of regional and global events with a broadening range of studies as more groups move into the field. Event monitoring has been carried out in a variety of settings including reservoir stimulation (e.g. [5,7,8]), geothermal seismicity [9], glacier icequakes [10,11] and urban monitoring [12–14]. Earthquake studies have used events at regional ranges [15–21], and out to teleseismic distances [22,23]. DAS recording has also been used in the analysis of ambient noise [12,18,24–27].

Some applications of DAS systems have deployed their own fibre optic cables (e.g. [10,16]), while many have exploited unused fibre channels on existing cables, termed ‘dark’ fibres (e.g. [18]). For specially deployed cable, the configuration is well controlled and shallow burial can be expected to give good ground coupling. For existing cables, although the general configuration may be known, cable loops in inspection pits can complicate the association of DAS channels with physical position. Also, for cables in a conduit the precise mode of deployment and the nature of the coupling to the surrounding environment can be difficult to determine. In urban environments, the ground coupling conditions can change quite rapidly along the cable with some modification of the DAS signal with position.

Because the nature and appearance of seismic ground velocity as recorded by seismometers is much more familiar than directional strain, many authors have made efforts to convert DAS strain-rate signals into equivalent ground velocity in different scenarios (e.g. [4,14,16,28,29]). The work of [29] suggests that a minimum of 20 gauge lengths of consistent cable will be needed to extract a reasonably accurate estimate of apparent wave velocity that is needed for the strain-rate conversion. However, the geometries of many cable layouts are not well suited to such conversion, with only short lengths in a consistent direction. Many ‘dark’ fibres, particularly in urban environments, have complex geometry linked to the configurations of streets with only short stretches of uniform orientation. This means that strain transformation is more difficult and so it is appropriate to work directly with the DAS system to understand the nature of the recorded seismic wavefield, which depends on both the detailed configuration of the DAS sampling and the orientation of the fibre relative to the components of the incoming seismic waves.

Here I discuss the nature of the seismic wavefield as seen directly by DAS recording on horizontal DAS cables taking account of the effects of gauge-length averaging of strain rate, and the geometrical configuration of the cable. Insight is provided by examining behaviour in the slowness–frequency domain for radially stratified models. I build on the results obtained by many previous researchers, but try to provide a unified treatment of the different aspects of the wavefield specific to DAS recording. Illustrations are provided of modelled DAS results for events close to a cable and for regional and teleseismic earthquakes.

The angular and slowness dependence of the DAS response means that the response of a DAS array is determined by the direction and epicentral distance of the event being imaged, rather than described by a general array function as in the case of seismometers. The DAS array response enhances slower travelling wave components such as surface waves compared with body waves with steeper paths through the near-surface zone.

## 2. Distributed acoustic sensing characteristics and seismic response

The sequence of laser pulses from the DAS interrogator are analysed to produce a measure of the relative change in optical path length Δg/g over a gauge length g that is determined by the nature of the laser pulses employed. Kushnikov [30] provides a detailed analysis of the changes in optical path length in terms of both fibre strain and the effects induced by the changes in dielectric effects under strain. For typical fibre optic properties, the change in optical path length is related to the axial strain along the fibre $\epsilon_{∥}$ and in the perpendicular direction $\epsilon_{⊥}$:

2.1 $$\frac{\Delta g}{g}\approx 0.7\epsilon_{∥}-0.2\epsilon_{⊥}.$$

For weak perpendicular strain $\epsilon_{⊥}$, the Poisson effect enhances the apparent longitudinal strain and so $\Delta g/g\approx 0.8\epsilon_{∥}$. Only when the axial strain is small relative to the tangential strain will the second term on the right-hand side of (2.1) become significant. This can occur when waves arrive broadside to the cable very close to a source.

The changes in optical path length around a channel location on the DAS cable are averaged over gauge length in the construction of the strain rate. The nature of the gauge length averaging depends on the pulse form employed in the DAS interrogation [1]. A consequence of the gauge length averaging, typically 10 m, is that a localized strong effect, such as that due to the immediate vicinity of a source, will affect a span of channels over a distance comparable to the gauge length. Such spatial averaging effects can be compounded by channel stacking procedures to enhance signal-to-noise ratio that are built into the DAS interrogator system, with different approaches employed by the various manufacturers. Such channel stacking helps to overcome optical fading issues associated with locally net zero optical scattering [2]. In general, spatial localization cannot be achieved to better than about half the gauge length, even when all channels are available.

Since DAS measurements are dominated by the strain along the optical fibre, they represent just a single component of the strain tensor. This means that the effect of a change in the direction of incoming waves by an angle θ is associated with tensorial strain rotation that depends on functions of 2θ rather than just θ for the vector rotation employed for the horizontal components of seismometers (e.g. [2,25]). As a result, the orientation of the optical fibre relative to incoming seismic waves plays an important role in determining the relative amplitude of DAS signals.

The DAS system renders the strain or strain rate along the optical fibre being sampled, but how far this represents the situation in the surrounding materials depends on the local coupling conditions (e.g. [18]). Such coupling issues are important in borehole situations since there is no natural clamping to the well wall. Where cable is specifically emplaced in trenches or glacier ice, coupling to the surroundings can be expected to be strong. Fortunately for horizontal near-surface cables in conduits, such as dark fibre, the effect of gravity and friction is generally sufficient to provide adequate linkage to the environment, though conditions can vary along a cable run. For cable in conduit, the geometrical configuration will depend on the nature of the conduit and cable loops may be introduced at inspection points to take up excess length.

A typical DAS cable lies at shallow depth, from just beneath the surface to 2 m, or so, depending on the particular configuration. This means that there is a slight time offset between up-going waves arriving at the fibre and the down-going waves reflected at the free surface. The delay is small, and can normally be neglected unless the surface wavespeeds are very low or very high frequencies are used with active sources.

As we have seen the relative change in optical path length due to disturbances passing across the fibre is dominantly controlled by the axial strain rate, which is just the spatial derivative of the ground velocity in the direction of the fibre $v_{d}$ with respect to distance along the fibre. Under the assumption of uniform sampling along the gauge length g, the effect of averaging the axial strain rate around the reference point takes the form

2.2 $$\langle {\dot{\epsilon }}_{d}\rangle =\frac{1}{g}\int_{-g/2}^{g/2}\text{d}s\;\frac{\partial v_{d}(s)}{\partial s}=\frac{1}{g}[v_{d}(s){]}_{s=-g/2}^{g/2}=\frac{1}{g}[v_{d}\left(\frac{g}{2}\right)-v_{d}\left(\frac{-g}{2}\right)].$$

**Figure 1.**
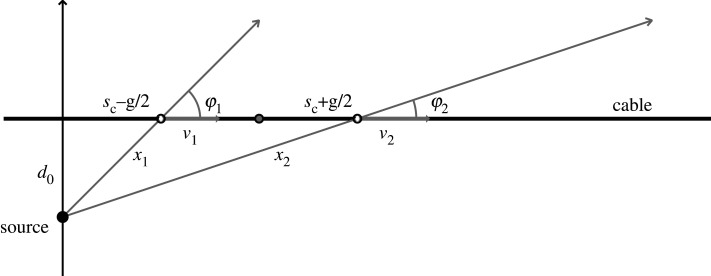
Plan view of a source near to a DAS cable. For a nominal cable channel at $s_{c}$ and source with closest distance to the cable of $d_{0}$, the velocity resolved along the cable $v_{1},v_{2}$ has to be calculated for the points $s_{c}\pm g/2$, which lie at different distances $x_{1},x_{2}$ from the source and with different inclinations of the radial vector to the cable $\varphi_{1},\varphi_{2}$.

The averaged strain rate can therefore, in principle, be obtained by differencing the ground velocity resolved along the cable at the ends of the gauge length. Such relations were first noted by Bakku [31] using a plane wave model for vertical seismic profiling, and have been widely employed for modelling DAS response for close sources (e.g. [4,8]) in borehole configurations. When an event is close to the DAS cable, the change in the waveforms across the gauge length can be significant due to the strong geometrical variations (figure 1) and so the appearance of the DAS wavefield does not directly correspond to what is seen in ground velocity on a geophone [32].

The effect of gauge length g is equivalent to imposing a spatial moving average [33] and, in consequence, imposes notches in the response for a suite of frequencies that are multiples of the inverse of the passage time of a wave across the gauge length [5,33], i.e. multiples of the apparent wave velocity along the cable divided by the gauge length. Such frequency variations are significant for the high frequencies employed in exploration work but should rarely impinge in seismological applications to natural events or site testing. The gauge length averaging also means that low-frequency waves with long wavelength are poorly recorded by DAS (e.g. [27]), because there is little variation across commonly used gauge intervals.

Wang *et al.* [16] have made a direct test of the relation (2.2) by constructing the difference of the output of two seismometers spanning the interval around a set of DAS channel locations for the frequency band 1–5 Hz at the Brady test site. Even though the distance between the seismometers spanned a number of channels, the summed DAS response over these channels was in good agreement with the difference between the ground velocity from the seismometers resolved along the cable direction. The configuration was equivalent to a rather long gauge interval.

For close events, the representation (2.2) can be used directly in modelling by differencing synthetic seismograms constructed from one-dimensional velocity profiles. For example, Hudson *et al.* [11] use reflectivity style calculations of Green’s functions for inversion of the mechanism of microseisms at the base of an ice stream in Antarctica. Wang *et al.* [16] show a few examples of calculated traces at regional distances produced by a similar approach. However, when the source lies hundreds of kilometres away from the cable the differences in event distance at the ends of the gauge interval are very small, and the ground velocities are very similar. As a result, there is a strong likelihood of significant loss of numerical precision when the velocity traces are differenced to produce strain rate. It is preferable for regional and teleseismic distances to work with analytic representations of the differencing within a slowness–frequency integration that can readily capture orientation effects along a complex cable geometry.

## 3. The distributed acoustic sensing seismic wavefield

Consider an array of DAS channels at locations $\left\{x_{c}\right\}$, and describe the cable configuration via the local unit tangent vector $d(x_{c})$, under the approximation of local linearity along the gauge length. This assumption generally works well except in the vicinity of a sharp turn in fibre direction.

A representation of the wavefield across the DAS cable can be built in terms of a expansion in plane waves in the slowness–frequency domain (e.g. [34]). For a position x the averaged strain rate along d(x) can be expressed as

3.1 $$\langle {\dot{\epsilon }}_{d}\rangle (\text{x},\omega )=\iint {\text{d}}^{2}\text{p}\langle {\dot{\epsilon }}_{d}\rangle (\text{p},\omega ){\text{e}}^{\text{i}\omega \text{p}\cdot \text{x}},$$

where p is the local slowness vector in the horizontal plane, with magnitude p. In terms of velocity differences (2.2), we have the alternative representation

3.2 $$\langle {\dot{\epsilon }}_{d}\rangle (\text{x},\omega )=\frac{1}{g}\iint {\text{d}}^{2}\text{p}\;[\text{v}\cdot \text{d}](\text{p},\omega )[{\text{e}}^{\text{i}\omega \text{p}\cdot {\text{x}}_{c+}}-{\text{e}}^{\text{i}\omega \text{p}\cdot {\text{x}}_{c-}}],$$

where $x_{c\pm }=x_{c}\pm gd/2$. The component [v⋅d](p,ω) is in common for the two ends of the gauge length and can be expressed in terms of the reflection and transmission properties of the medium. With the introduction of a source at the origin, the double integration over horizontal vector slowness p can be recast as an integral over slowness p accompanied by an angular expansion in vector harmonics (e.g.[34]) as in the usual approach to synthetic seismograms for a layered medium. The differencing associated with the gauge length means that it is necessary to take account of the differences in range and azimuth relative to the DAS cable at the two ends. As illustrated in figure 1, for sources close to the cable line there can be noticeable differences in these quantities.

Interference effects from shallow cable emplacement only become significant at high frequencies, and can be included by replacing the free-surface reflection coefficients that depend only on slowness by a frequency-dependent term representing the interaction of up-going and down-going waves at the cable depth.

### (a) Close sources

The relation (2.2) means that, in principle, we can work directly with existing ways of calculating synthetic seismograms, but need to include the differencing effects of the gauge length. This can be done by explicit differencing of prior ground velocity calculations provided that the geometrical configuration is fully specified (e.g. [11,16]). The alternative is differencing while constructing the synthetics in the slowness–frequency domain as in (3.2), which has the advantage that many channels can be calculated exploiting the same medium response terms once the cable configuration has been mapped. Direct differencing is most effective for close sources to the DAS fibre. Once the source lies well away from the cable so that the arriving wavefronts are approximately plane, the accuracy of differencing is reduced with loss of numerical precision.

In the examples in §4, DAS synthesis is implemented in the frequency–slowness domain using the approach described in [35] that builds the response of the multi-layered model in terms of the reflection and transmission properties of the layers, with the inclusion of surface force sources as well as internal moment tensor sources. For close sources a direct differencing approach is employed in the slowness–frequency domain. For more distant sources an analytical representation of the differencing operator is applied, as detailed in the section below.

### (b) Distant sources

When the DAS cable lies well away from the source of seismic waves the three-component ground velocity field has similar character across even a kilometre or two of cable. The main effects now come from the orientation of the cable with respect to the incident arrivals. With frequency–slowness synthesis, we build the response from a spectrum of plane waves, and can develop a formalism for the effect of gauge length on distant events. I here present a general formulation for a horizontal DAS cable building on the analysis in the supplementary material to [10].

Consider a plane wave with frequency ω that has propagated a distance Δ to a reference point from a distant source with horizontal slowness vector p. In the neighbourhood of the reference point, the displacement associated with the plane wave

3.3 $$\text{u}=u\text{n}{\text{e}}^{\text{i}\omega [\text{p}\cdot \text{x}+p\Delta ]},$$

where x is the offset from the reference point and n is the polarization vector for the plane wave. The associated strain tensor

3.4 $$\epsilon_{ij}=\frac{1}{2}\left(\frac{\partial u_{i}}{\partial x_{j}}+\frac{\partial u_{j}}{\partial x_{i}}\right)=\frac{\text{i}\omega }{2}u(n_{i}p_{j}+n_{j}p_{i}){\text{e}}^{\text{i}\omega [\text{p}\cdot \text{x}+p\Delta ]}.$$

The strain resolved along a DAS cable lying in the local tangent direction d is then

3.5 $$\epsilon_{d}=d_{i}\epsilon_{ij}d_{j}=\text{i}\omega u(\text{d}\cdot \text{n})(\text{d}\cdot \text{p}){\text{e}}^{\text{i}\omega [\text{p}\cdot \text{x}+p\Delta ]}.$$

The DAS measurement associated with a point represents an average of strain along the gauge length g along the cable around that point. Thus, setting x=sd aligned along the local cable direction, the averaged strain around the reference point

3.6 $$\begin{array}{rl} \left\langle \epsilon_{d}(\omega )\right\rangle & \;=\frac{1}{g}\int_{-g/2}^{g/2}\;\text{d}s\;\epsilon_{d}(s)=\frac{1}{g}u(\text{d}\cdot \text{n})[{\text{e}}^{\text{i}\omega [s\text{d}\cdot \text{p}+p\Delta ]}{]}_{s=-g/2}^{g/2}\\ & \;=\frac{2\text{i}}{g}u(\text{d}\cdot \text{n})\sin(\frac{\omega g}{2}\text{d}\cdot \text{p}){\text{e}}^{\text{i}\omega p\Delta }. \end{array}$$

For systems that record strain rate, such as the iDAS, the averaged strain rate is

3.7 $$\langle {\dot{\epsilon }}_{d}(\omega )\rangle =\frac{2\omega }{g}u(\text{d}\cdot \text{n})\sin(\frac{\omega g}{2}\text{d}\cdot \text{p}){\text{e}}^{\text{i}\omega p\Delta }.$$

The term d⋅n depends on the polarization. The combination $p_{d}=d\cdot p$ represents the slowness of the plane wave resolved along the cable, and so $p_{d}=p\cos\psi$ as a function of the inclination of the cable ψ relative to the radial direction from the source.

Relations equivalent to (3.7) have been derived in many ways with differing notation, commonly employing the effective propagation velocity along the cable $c_{d}=1/p_{d}=1/(p\cos\psi )$. For high-frequency waves, the full form of (3.7) is needed, and has been applied by Egorov *et al*. [28] to convert averaged strain to ground velocity for vertical seismic profiling so that they can employ standard methods for full waveform inversion.

When the variation of the plane wave along the cable is slow compared to the gauge length, i.e. $\omega gp_{d}/2\ll 1$, the sine in (3.7) can be approximated by its argument. In this case,

3.8 $$\langle {\dot{\epsilon }}_{d}(\omega )\rangle =\omega^{2}u(\text{d}\cdot \text{n})(\text{d}\cdot \text{p}){\text{e}}^{\text{i}\omega p\Delta },$$

which is the acceleration due to the plane wave resolved along the cable modulated by the slowness along the cable. The averaged strain rate $\left\langle {\dot{\epsilon }}_{d}\right\rangle$ can thus be constructed by combining the radial $u_{r}$ and tangential $u_{t}$ components as

3.9 $$\langle {\dot{\epsilon }}_{d}(\omega )\rangle =\omega^{2}p\cos\psi (u_{r}\cos\psi +u_{t}\sin\psi ){\text{e}}^{\text{i}\omega p\Delta }.$$

This approximation will be suitable for frequencies

3.10 $$f<f_{c}=\frac{1}{\left(5\pi gp\right)},$$

since the strongest variation occurs when the cable and propagation path are aligned. For regional phases with a gauge length of 10 m, $f_{c}∼40\;\text{Hz}$ for *P* waves and $f_{c}∼$ 20 Hz for *S* waves. The values of $f_{c}$ are even higher for teleseismic waves, with a steeper inclination to the vertical and so smaller horizontal slowness.

The approximate result (3.9) does not depend on the precise nature of the averaging along the gauge length. The relationship between averaged strain and ground velocity $v_{d}$ projected along the cable is often given in the apparently simpler time domain form (e.g. [4]):

3.11 $$\langle \epsilon_{d}\rangle =\frac{v_{d}}{c_{d}},$$

and forms the basis of conversion schemes to extract ground velocity from DAS records for sources lying well away from the cable (e.g. [16,22,23]).

The approximation (3.9) is suitable for both regional and teleseismic distances, and can be implemented by simple modifications of the receiver terms in the frequency–slowness response before integration. For synthesis using modal summation, the DAS response can again be obtained by a modification of the receiver terms; for a mode with frequency ω and angular order l the slowness p=ω/(l+1/2). To apply (3.9) the source needs to be sufficiently far from the array for the local field to appear as a passing plane wave, so that a minimum distance of a few tens of kilometres is needed for natural sources. Nevertheless, the inclination factors can be applied for closer sources and provide a convenient rapid assessment of response.

The DAS response term (3.9) applies a factor of horizontal slowness to acceleration, irrespective of the relative orientation of the cable and the incoming wave. This means that steeply arriving waves with small slowness, such as P and S body waves from distant sources, are diminished, while the late surface waves with large slowness are amplified. As a result, Rayleigh waves are even more pronounced on broad-band DAS records of distant events than on comparable seismograms [22], and the surface wave coda is extended. The amplification of locally scattered surface waves can also have the effect of reducing the coherence of DAS recording of body waves [20].

When the condition (3.10) is satisfied there is simple scaling between projected ground velocity and averaged strain rate linked by the effective slowness along the fibre. This relation has been used by a number of authors (e.g. [16,22]) to implement conversion to ground velocity using *f*–*k *mapping, but such conversion requires a significant length of fibre with a common orientation, and needs careful regularization when data have only a narrow wavenumber content. An alternative is to estimate the apparent slowness for the portion of the records being considered; e.g. Paitz *et al.* [23] have used Rayleigh wavespeeds for the surface wave portions of recordings in Switzerland of events in Greece and Fiji.

As noted by Walter *et al.* [10] the action of the DAS strain-rate averaging in (3.9) is to change the amplitude spectrum of the wavefield, while leaving the phase associated with propagation from the source unchanged. This means that travel-time picking is unaffected by the DAS recording system provided the frequency is not too high.

### (c) Orientation factors

The net result of the tensorial projection of strain onto the oriented cable is that the behaviour is controlled by the relative angle ψ between the passing wavefront and the DAS cable. For a horizontal cable, the radial component of acceleration from P and SV waves has the projection factor

3.12 $$u_{r}\cos^{2}\psi =\frac{u_{r}(\cos2\psi -1)}{2},$$

and for the tangential component from *SH* waves

3.13 $$u_{t}\sin\psi \cos\psi =\frac{u_{t}(\sin2\psi )}{2}.$$

The radial contribution is always positive, while the transverse contribution can change sign. The double angle dependence comes from the tensor projection of strain. Martin [25] provides a detailed discussion of orientation effects for different classes of waves, both for direct measurement and for cross-correlation as in the analysis of ambient noise.

For Rayleigh waves, the radial orientation term $\cos^{2}\psi$ is to be applied, with maximum response along the DAS cable (e.g. [2,25]). For Love waves, the tangential factor sin⁡ψcos⁡ψ comes into play and this 4-lobed pattern has nulls for waves travelling along and perpendicular to the DAS cable. As a result, Rayleigh waves tend to be more prominent on DAS recordings than Love waves (e.g. [22]).

Since DAS records are commonly plotted in terms of distance along cable without consideration of orientation factors it is convenient to have a summary of such effects. This can be provided by plotting out the orientation terms on the same scale as the record section. Examples are shown below for regional and teleseismic events across arrays with notable changes of geometry. Such diagrams can be helpful in disentangling effects arising from the interference of different phases from changes in direction of the cable layout.

## 4. Examples of distributed acoustic sensing wavefields

In this section, I show how the nature of the DAS response discussed above can be used to provide understanding of the character of the strain field and to model DAS records directly. I present examples for horizontal DAS cables. In addition to the gauge length, a number of other choices, e.g. for channel stacking, can be imposed by the DAS acquisition systems and these can modify the appearance of the strain-rate records. For simplicity, I have chosen to extract single channel DAS responses in the simulations that could be post-processed to provide a direct match to specific DAS interrogator settings.

To be able to model DAS records, we must find (i) a suitable source representation, (ii) an appropriate propagation model and (iii) a detailed description of the configuration of the channels along the optical fibre in terms of position and orientation. The geometry of the cable can be well specified when a specific deployment has been made. But, for dark fibres in existing telecommunications systems, although the general pattern of cable run may be known, some effort may be needed to delineate the channel configuration, e.g. using localized sources (tap tests) to calibrate channel count against position. The distance along the ground is commonly shorter than the distance along the fibre being interrogated, due to slack in the fibre deployment. Additional effects can also arise where cable loops are included at access points.

A suitable description of a local source will normally have a strong vertical component, but a jump or a sledgehammer blow will commonly have some horizontal effect as well. For regional and teleseismic events, source information for larger events can be found from various agencies as fault plane mechanisms or full moment tensors, but smaller events may be poorly characterized from few recordings.

Successful simulation using simple one-dimensional models requires that the conditions in the neighbourhood of the DAS cable layout do not show too much variation. Coupling of the DAS cable to its environment can vary noticeably, leading to amplitude discrepancies that could be ameliorated by post-processing of the model results, if sufficient information is available. One of the merits of direct DAS modelling is that in areas within consistent local structure the model results can reveal variations in cable coupling by comparison of expected and observed amplitude patterns. Localized variations in the nature of the DAS signal can also be produced by variations in structure (e.g. [27]) and can be difficult to separate from coupling issues. With poor coupling the observed signal is attenuated, but will tend to change shape under the influence of structure.

### (a) Local sources

The applications of DAS recording in exploration contexts have the advantage of well-calibrated cable configurations and controlled sources so that high frequencies can be effectively exploited (e.g. [4,8]). However, where existing telecommunication cables are being exploited only the basic geometry of the path is well known but not the mapping of individual channels.

As part of the procedure for calibrating the channel locations along a dark fibre it is common to carry out tap tests with a localized source, e.g. a sledgehammer blow or a person jumping. The strongest effects should then appear at the closest DAS channel. The source point will normally lie close to the cable, but not right on top, and so the offset from the cable and the consequent variation in the rotation of the velocity field to lie along the cable need to be taken into consideration when modelling the DAS records by differencing (figure 1).

**Figure 2.**
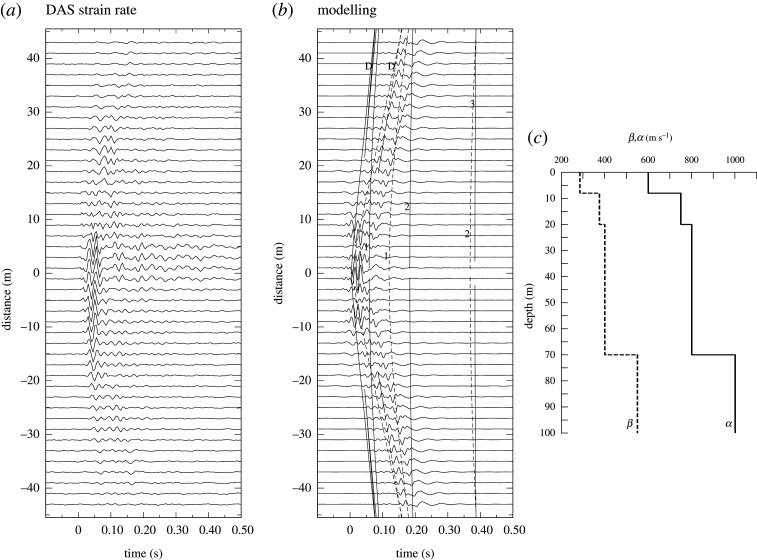
A jump test in a street in Bern, Switzerland: gauge length 10 m, channel spacing 2 m. (*a*) Strain rate observations. (*b*) Simulation by velocity differencing in the slowness–frequency domain. Travel time calculations for a source displaced 2 m from the cable are shown in the left-hand panel, to help identify the arrivals. P waves are shown in dark grey and S waves with dashed lines; *D* indicates the direct and refracted arrivals, the numbers indicate reflection from the interfaces. (*c*) Wavespeed depth profile for P (solid) and S (dashed).

The character of the results for tap tests depends strongly on the local conditions, and the proximity of the source to the cable whose actual location may well not be known. Generally there is a strong localized response, often from S waves. In favourable circumstances, the DAS recordings show the equivalent of a refraction spread that can be used to extract information on near surface structure (as in figure 2). In the immediate vicinity of tap tests, DAS records on dark fibre are often complex with long duration, and this could be associated with wave trapping in the cable conduit.

As an example of a test in an urban environment, figure 2 illustrates a single jump on the sidewalk of a street in Bern, Switzerland recorded on an optical fibre connecting educational institutions about 2 km apart (A. Fichtner, D. Bowden and K. Smolinski 2021, personal communication). The cable lies at a depth of 0.7 m, with relatively good coupling to the surroundings. The gauge length employed was 10 m, and DAS channels were extracted at 2 m intervals. In this case, the jump energy has propagated to considerable distance and there is notable energy in refracted P.

The segment of data illustrated in figure 2 lies along a straight section of the street, but the nature of the buildings changes along the profile, including the presence of basements with concrete walls that can act as side reflectors. Such differences along a street are common in an urban environment and mean that the pattern of observations is not symmetric about the closest channel. Thus, any one-dimensional model will only be an approximation to the actual situation and a tight match to observations cannot be expected.

The modelling shown in figure 2*b* uses the simple layered model shown in figure 2*c* that gives a reasonable representation of the major arrivals at a number of different jump points and is consistent with local surface wave dispersion results. The effect of a person jumping has been simulated with a strong vertical force and much weaker horizontal components. The exact position of the jump point relative to the cable is not known, so I have assumed a separation of 2 m from the cable for the simulation. The synthesis used 2500 slownesses out to $4\;\text{s}\;{\text{km}}^{-1}$, for an 0.8192 s time interval with 4096 time points. The pass band was 0.25 to 55 Hz and moderate attenuation was included in the model with $Q_{p}^{-1}=0.01$, $Q_{s}^{-1}=0.02$. The travel time curves plotted in figure 2 on the synthetics are calculated including the effect of the offset source, but do not allow for the mixing effect from velocity differencing at the ends of the gauge length that is included in the seismograms. A consequence of differencing is that a single DAS trace can mix aspects of the wavefield with different character, e.g. refracted and reflected segments with similar timing separated by the gauge length. Such an effect was noted by Zhu *et al.* [14] in a comparison of a spread geophone and DAS records close to a hammer blow. Near the source some cancellation can occur from differencing. At larger offsets the main effect is simple spatial averaging.

No channel stacking has been applied to the modelled results in figure 2. However, it would appear that a five-channel running average has been applied to the observations (thus spanning 10 m the same as the gauge length). This gives the impression of a more localized response but at the expense of further smearing of the features of the wavefield. It is therefore necessary to investigate closely the settings employed in any DAS deployment if a full simulation is to be made.

**Figure 3.**
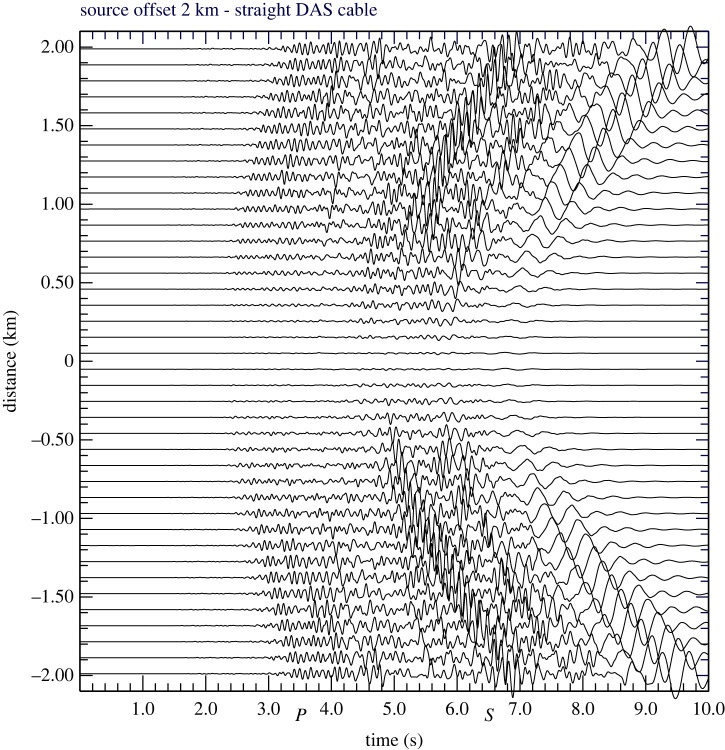
Modelling for a shallow source at 2 km from a straight cable showing the bland zone for strain rate on the section of cable closest to the source.

A consequence of the inclination factor (3.7) is that P waves at near normal incidence have very low amplitude in DAS records. This means that on the section of the cable in the closest proximity to a source lying some distance away there will be a rather bland zone with low amplitude arrivals extending either side of the closest approach of the cable to the source. An example is shown in figure 3 for a moment tensor source with dominant $M_{zz}$ component at 40 m depth, 2 km away from a straight cable using the same wavespeed model as in figure 2*c*. The span of distances for which the inclination is close to normal increases with source displacement but the gradient in amplitude becomes weaker and the effect is less obvious. A similar feature is seen in fig. 3 of [5] with close observation of a microseismic event on a horizontal monitoring cable at depth. A comparable problem of reduced response to P waves at the closest distances occurs for sources lying beneath a DAS array. The P waves are suppressed for steep paths with small horizontal slowness p (e.g. [11]). Sensitivity to P waves can be ameliorated by special cable construction with engineered fibre, e.g. the helical winding analysed by Kuvshinov [30] and Wuestefeld & Wilks [36].

### (b) Regional sources

As an example of the high-frequency component of the regional wavefield, I show a recording of a regional earthquake at the Tidbinbilla Deep Space Tracking Station south of Canberra in Australia (V. Lai, M. S. Miller and H. McQueen 2021, personal communication). A spare fibre leading from the central building to the north to one of the antennas was used for the DAS system. The cable lies at a depth of 2 m and the path follows a set of straight-line segments with some sharp changes of orientation. The active operations at this site generate significant low frequency noise, which limits the usable passband to above 1 Hz. This means that the DAS data lie in the regime where influences from scattering are strong, and the coherence of signals is reduced.

**Figure 4.**
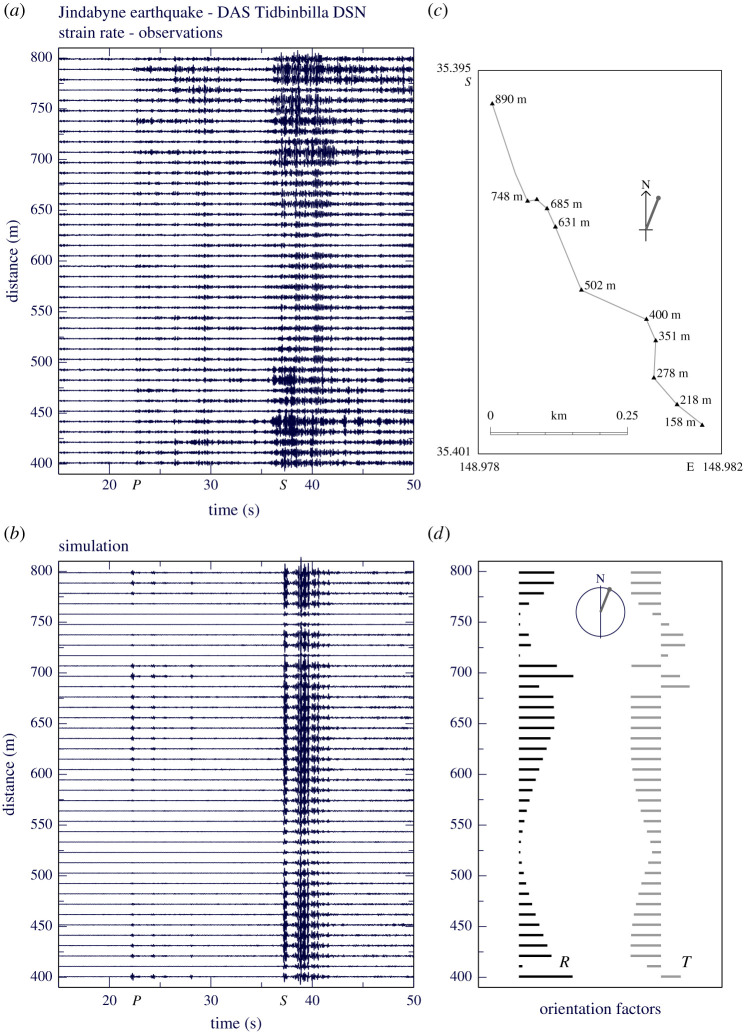
Regional event: Jindabyne earthquake ML 3.7 of 5 May 2021 recorded at the Tidbinbilla Deep Space Tracking Station south of Canberra, Australia. The data are high-pass filtered above 1 Hz to eliminate strong site noise. (*a*) Strain rate observations for the DAS cable, filter 1.0–30.0 Hz; (*b*) DAS modelling using a slowness–frequency integration; (*c*) configuration of the DAS cable; (*d*) orientation factors for P, *SV* waves (R) and *SH* waves (T), with the azimuth of the arriving wavefront indicated—distance scale in metres as in (*b*). (Online version in colour.)

Figure 4*a* illustrates the recordings from an event to the south at a distance of 125 km crossing the cable with azimuth $21.5^{\circ }$. Modulation of the amplitude of the observed arrivals and changes in the structure of the waveforms can be linked to the varying orientation of the segments of the cable (figure 4*c*,*d*). The cable configuration was calibrated by tap tests. There are complications in the geometry around 700 m cable distance where an excess cable loop has been introduced. This loop probably accounts for the rapid changes in recorded signal near this location (figure 4*a*). The surface conditions vary, with ground disturbance in places associated with past building work.

The synthetics displayed in figure 4*b* were calculated using a regional velocity model with a gradient zone at the base of the crust from 35 to 45 km. The moment tensor source, based on previous events in the area, was set at 6 km depth since this provided the best representation of wavefield character. The azimuth to the DAS array lies close to a node in the P wave radiation pattern, so that S waves are enhanced by both scaling by horizontal slowness and the radiation pattern effects. The path from the source crosses a crustal region with pronounced wavespeed gradients (e.g. [37]), and thus it is hard to find a suitable wavespeed model for high-frequency simulation. For the modelling, the DAS strain rate simulation was carried out using the mapping of acceleration (3.9) onto the cable layout (figure 4*c*). The orientation factors for the radial and tangential components of strain are displayed in figure 4*d* alongside the synthetics on the same distance scale, so that their influence can be judged.

Slowness integration was taken out to $0.33\;\text{s}\;{\text{km}}^{-1}$ with 1200 slownesses and a frequency passband from 0.1 to 8.0 Hz. A modest amount of signal generated noise has been added using the approach described by Kennett [38] in which a random component modifies the frequency–slowness spectrum. The synthesized regional seismograms are still too simple, but the pattern of visibility of the *P* and *S* arrivals shows a reasonable correspondence with the observations. The synthetics only include propagation along the great-circle path, but because the azimuth is close to a P wave radiation node, scattering effects from off path have a significant influence (as seen in the observations). The reduced S wave amplitude between 500 and 650 m can be linked to a change in ground conditions in the material surrounding the conduit. Such an effect could not be recognized without the aid of the model.

### (c) Teleseismic sources

For events at teleseismic distances, the dominant contribution on DAS records at low frequencies will be surface waves, usually dominantly Rayleigh waves, enhanced by the amplification of acceleration by horizontal slowness (e.g. [22,23]). At higher frequencies, in favourable circumstances, it can be possible to pick up teleseismic arrivals on dark fibre in urban areas.

**Figure 5.**
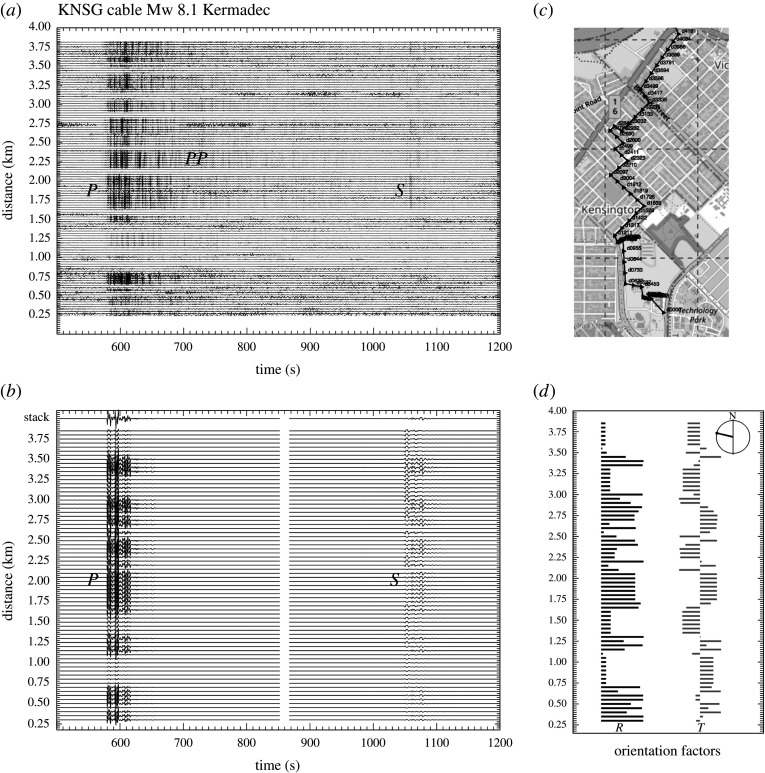
Teleseismic event: Kermadec earthquake Mw 8.1 of 4 March 2021 recorded on an urban cable in Perth, Western Australia. (*a*) Strain rate observations for the DAS cable, filter 0.1–1.0 Hz; (*b*) DAS simulation using a slowness bundle around the geometric slowness, the S portion is shown at higher amplification for clarity; (*c*) configuration of the illustrated portion of the cable, superimposed on Open Street Map; (*d*) orientation factors for P, *SV* waves (R) and *SH* waves (T), with the azimuth of the arriving wavefront indicated—distance scale in kilometres as in (*b*).

Figure 5*a* displays the recording of the Mw 8.1 earthquake in the Kermadecs (4 March 2021, $29.723^{\circ }\text{S}$, $177.279^{\circ }\text{W}$, 30 km depth) on an urban cable in Perth, Western Australia connecting CSIRO establishments (E. Saygin and L. Ricard 2021, personal communication). The cable run follows the pattern of streets and includes many rapid changes in direction (figure 5*c*). The event lies at $56.5^{\circ }$ away from the cable, with takeoff azimuth at the source of $249.4^{\circ }$. The wavefield crosses the DAS array at an azimuth of $286.6^{\circ }$. A consequence of the changes in orientation of the cable relative to the arrival direction from the source (figure 5*c*) is that there is distinct banding in the amplitude of the observations, as well as variations that can be linked to changing ground coupling (e.g. in the street segment from 1.00 to 1.25 km).

To model the teleseismic body waves the approach of [39] has been used. This approach includes crustal response at both source and receiver sides coupled to a simple reflection from the mantle with allowance for attenuation. A slowness–frequency integration is performed over a bundle of slownesses around the ray theoretical value, which allows full modelling of the direct phases *P*, *S* and their interactions with their associated depth phases.

In this case, the *ak135* model [40] was used for both source and receiver crustal structure with $Q_{p}^{-1}=0.001$, $Q_{s}^{-1}=0.002$. The mantle model was also *ak135* with the loss factors increased to $Q_{p}^{-1}=0.005$, $Q_{s}^{-1}=0.001$ between 120 and 660 km depth. The CMT solution for the event was used with a point source at 29 km depth. A bundle of 120 slownesses around the geometrical ray value was used separately for the *P* and *S* arrivals with a frequency band from 0.05 to 1 Hz. A trapezoidal wavefunction 5 s long was employed with rise and decay times of 1.25 s. Because the stations along a DAS cable are closely spaced the geometric slowness varies slowly. This means that the same bundle of slownesses can be employed in modelling for a sweep of stations at a time, thereby reducing computational effort significantly.

The modelled DAS records (figure 5*b*) reproduce well the modulation of the *P* arrivals by orientation, and there is a hint of the shift in emphasis between the direct and depth phases seen in the observations. The complex pattern of orientation effects due to the changes in cable direction are shown in figure 5*d* on the same distance scale as the synthetics and observations. Some slight shifts in the position of amplitude minima between the observations and the modelling could be a product of an imperfect mapping of channel position.

Both the simulation and the DAS observations show reasonable consistency across the array in the style of waveforms, even though the amplitudes are affected by orientation. At the top of the simulation panel (figure 5*b*), I show the stacks of the *P* and *S* wave traces, normalized by the number of traces. For the *P* waves, the stack trace provides a good rendering of the character of the arrivals with little distortion. However, for *S* there are significant variations in polarity of the contribution from the *SH* waves, on the transverse component to the path from the source. These are most noticeable at the onset of *S* on the DAS simulations. Their net effect on stacking is to almost entirely suppress the onset of *S* in the stack trace leaving only modest depth phase contributions from the radial component (*SV*).

The significant amplitude difference between the modelling and the observations around 1.25 km indicates a segment along a road with very different coupling conditions from the rest of the cable. Although the synthetics indicate little change in P waveform along the cable, there are some distinct shifts in the pattern in the observations consistent with a higher emphasis on the depth phases. There is strong coherence over road segments of up to 400 m, but changes along different roads that may be associated with the nature of the cable conduit.

The success of the relatively simple modification of the teleseismic response with the slowness and angular weight to simulate DAS records suggests that waveform inversion for source characterization will be feasible. With stacking of a number of DAS channels on a well-oriented cable segment, to increase the signal-to-noise ratio, the DAS results could be used directly in an inversion scheme, such as that described by Kennett *et al*. [41], along with seismometer records.

## 5. Distributed acoustic sensing array response

The capacity to provide a direct simulation of DAS signals opens the way to using such results for planning layouts for experimental deployments of optical fibre cables, including the influence of cable orientation and wave type.

We have seen in figure 5 that direct stacking of DAS signals can be effective for *P* waves, even for a cable configuration with complex changes of orientation. Can such an approach be carried further and a DAS cable configuration be used for signal analysis using array processing? The very high number of available DAS channels would suggest that it should be possible to use even a modest size cable layout to enhance signal at higher frequencies. Yet, the use of DAS recording brings in a dependence of the recorded response on the orientation of the cable with respect to the passage of the seismic wavefield that does not arise for seismometers, where rotation of components is available. As a result, the process of stacking an array of DAS channels has a somewhat different character than for an array of seismometers. Such effects have been noted previously (e.g. [20,22]) in empirical analysis of DAS stacking.

Array processing is designed to enhance coherent signal crossing an array by making suitable combinations of the traces. Distant events can be represented by a dominant plane wave with slowness s. Thus, adjusting the timing of the records from the different array sites to compensate for the expected phase differences and combining the set of shifted traces, arrivals with slowness close to s will be enhanced since these will be coherent across the set of traces.

The linear array sum for an incident plane wave, with appropriate time delays at the N DAS channels, takes the form

5.1 $$E(\text{s},\omega )=\frac{1}{N}\sum_{j=1}^{N}\langle \dot{\epsilon }\rangle ({\text{x}}_{j},\omega ){\text{e}}^{-\text{i}\omega [{\text{x}}_{j}.\text{s}]},$$

in terms of the vector slowness s and the sensor coordinates $x_{j}$ relative to a reference site at a suitable origin. For a single plane wave travelling with an azimuth ϕ, and an inclination to the vertical i, the horizontal slowness vector s=s(cos⁡ϕsin⁡i,sin⁡ϕsin⁡i).

For a distant event, from the representations (3.8) and (3.9) we can express the ground motion at frequency ω crossing a DAS array as a superposition of plane wave components

5.2 $$\langle \dot{\epsilon }\rangle (x_{1},x_{2},\omega )=\omega^{2}\int_{-\infty }^{\infty }\int_{-\infty }^{\infty }\text{d}p_{1}\text{d}p_{2}(\text{d}(\text{x})\cdot \text{n})(\text{d}(\text{x})\cdot \text{p})u(p_{1},p_{2},\omega ){\text{e}}^{\text{i}\omega [p_{1}x_{1}+p_{2}x_{2}]},$$

where u(p,w) is the displacement recorded by a seismometer and d(x) is the local unit vector tangent to the cable. With this form for $\left\langle \dot{\epsilon }\rangle (x_{j},\omega \right)$, the array sum (5.1) becomes

5.3 $$\begin{array}{rl} E(\text{s},\omega ) & \;=\sum_{j=1}^{N}\omega^{4}\int_{-\infty }^{\infty }\int_{-\infty }^{\infty }\text{d}p_{1}\text{d}p_{2}u(p_{1},p_{2},\omega )(\text{d}({\text{x}}_{j})\cdot \text{n})(\text{d}({\text{x}}_{j})\cdot \text{p}){\text{e}}^{\text{i}\omega [(p_{1}-s_{1})x_{j1}+(p_{2}-s_{2})x_{j2}]}\\ & \;=\omega^{4}\int_{-\infty }^{\infty }\int_{-\infty }^{\infty }\text{d}p_{1}\text{d}p_{2}\;u(p_{1},p_{2},\omega )\sum_{j=1}^{N}(\text{d}({\text{x}}_{j})\cdot \text{n})(\text{d}({\text{x}}_{j})\cdot \text{p}){\text{e}}^{\text{i}\omega [(\text{p}-\text{s})\cdot {\text{x}}_{j}]}, \end{array}$$

where the sum involves both the polarization and the orientation of the cable for each plane wave component in the wavefield. The time shifts depend on the differential slowness p−s, but the orientation factors depend solely on p.

This complex result for a DAS array is in strong contrast with the situation for an array of seismometers where the wavefield is just modulated by the array response function

5.4 $$S(\Delta \text{s},\omega )=\sum_{j=1}^{N}{\text{e}}^{\text{i}\omega [\Delta \text{s}.{\text{x}}_{j}]},$$

shifted to the differential slowness Δs=p−s. For seismometers, the same functional form is derived irrespective of the slowness s and the pattern in slowness space is dictated by the geometry of the array. S(Δs,ω) is a scaled version of the Fourier transform of a set of delta-functions placed at the array positions with respect to wavenumber. The function S(Δs,ω) can be characterized by calculating the response for a vertically incident wave for which $s_{1}=s_{2}=0$.

The direct array response for the DAS cable modulates the seismometer array response by the slowness, so the specific behaviour depends on the nature of the incident wavefront. There is almost no response for vertical incidence when the wave motion is perpendicular to the cable, but sensitivity grows as the angle of incidence to the vertical increases. From (3.9) the orientation factor associated with the radial component of motion to the path from the source (*P* and *SV* waves) is $\cos^{2}\psi_{j}$, where $\psi_{j}$ is the angle between the local slowness vector $p=(p_{1},p_{2})$ and the tangent to the cable at each site $x_{j}$. This factor will always be less than unity and thus stacking will tend to be less effective than for the similar configuration of seismometers even with amplification by the magnitude of the horizontal slowness. For the transverse component of motion (*SH* waves) the orientation factor is $\cos\psi_{j}\sin\psi_{j}$ and so can take both positive and negative values. The net result is that stacking does not focus on the target slowness, as we have seen in the *S* stack for the teleseismic event in figure 5.

**Figure 6.**
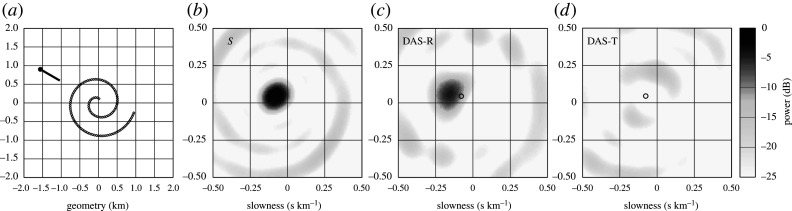
Simulation of array response for an Archimedean spiral including DAS cable orientation effects at 4 Hz. (*a*) Array geometry for 6 km cable with an incident wavefront crossing the array with an azimuth of $300^{\circ }$ and slowness 10.0 s per degree for an event at epicentral distance $20.3^{\circ }$. (*b*) Array response with no allowance for orientation (S), equivalent to seismometer stack. (*c*) Array response including orientation effects for radial motion (DAS-R: for *P-SV* waves). (*d*) Array response including orientation effects for transverse motion (DAS-T: for *SH* waves). The response is shown as relative power to the peak of the array response in (*b*), with the colour scale shown at the right. The location of the true slowness vector is indicated with an open circle in (*b–d*).

A very suitable configuration for creating an array from a DAS cable is an Archimedean spiral with polar equation r=aθ, which closely resembles a set of concentric circles with gentle curvature using a single continuous fibre (figure 6*a*). Such a spiral has been shown to have good characteristics with even a limited number of available sensors [42,43]. Here, for simplicity, I consider a 6 km long cable (a=80 m) with DAS channels extracted every 50 m, to give a total of 120 effective sensors along the spiral. The stack response of the DAS array depends on the specifics of the incoming wavefront. In figure 6, the wavefront comes from a far regional event at 4 Hz with a horizontal slowness of 10 s per degree, i.e. an apparent velocity of $11.2\;\text{km}\;{\text{s}}^{-1}$. For a surface P wavespeed of $4\;\text{km}\;{\text{s}}^{-1}$, this would correspond to an incidence angle of $21^{\circ }$ for P and an incident angle of $12^{\circ }$ for S. The array power distribution is constructed as a function of slowness for both a set of seismometers with the same spatial configuration (figure 6*b*) and the DAS array for the radial and tangential components from the source (figure 6*c*,*d*). Even with the modest size array, the number of sensors means that it possible to achieve strong targeting without any orientation effects. Side bands are weak and well separated from the main response. Once the DAS orientation effects are included (figure 6*c*) there is still a good concentration of the array response around the correct azimuth, but the peak is displaced to larger slowness (as would be appropriate to a closer event). Hence the DAS cable has a useful array response for *P* and *SV* waves that register on the radial component from the source, provided the event is not too far away. For the transverse component (*SH* waves), the orientation terms kill off the response at the target slowness and weak lobes lie to the sides of radial peak (figure 6*d*).

**Figure 7.**
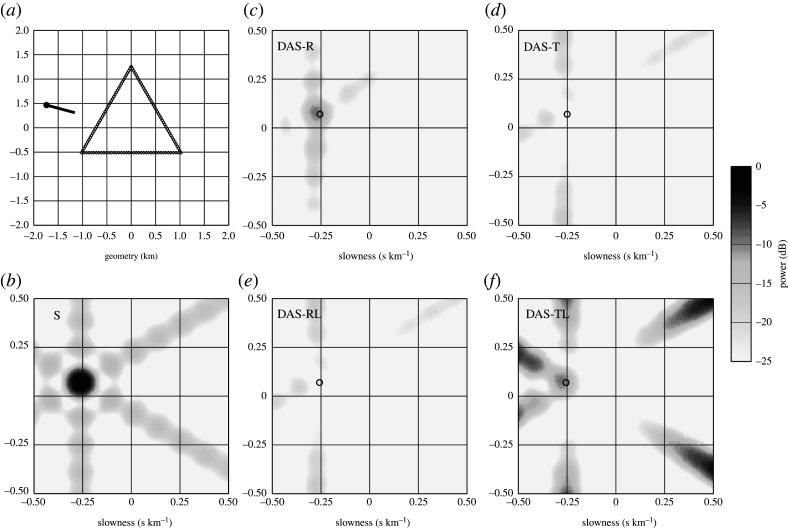
Simulation of array response including DAS cable orientation effects at 4 Hz, for a triangular array with enhanced stacking of *SH* component. (*a*) Array geometry for 6 km cable with an incident wavefront crossing the array with an azimuth of $285^{\circ }$ and slowness 30.0 s per degree. (*b*) Array response with no allowance for orientation (S), equivalent to seismometer stack. (*c*) Array response including orientation effects for radial motion (DAS-R: for *P-SV* waves). (*d*) Array response including orientation effects for transverse motion (DAS-T: for *SH* waves). (*e*) Modified array response to suppress radial motion (DAS-RL: for *P-SV* waves). (*f*) Modified array response to enhance transverse motion (DAS-TL: for *SH* waves). The response is shown as relative power to the peak of the array response in (*b*), with the colour scale shown at the right. The location of the true slowness vector is indicated with an open circle in (*b–f*).

For surface waves, which already have a large slowness, the distortion introduced by the DAS array stacking is much less, and so stacking for Rayleigh waves on the radial component can be expected to work well (figure 7*c*: DAS-R), but Love waves on the tangential component will be suppressed (figure 7*d*: DAS-T). The simple triangular array has stronger sidelobes than the spiral in figure 6. Luo *et al.* [44] have demonstrated that Rayleigh and Love wave separation can be achieved by using orthogonal arms of DAS cable with addition and subtraction of the contributions from the arms. Their idea can be generalized to other array configurations.

We have noted above (3.12) and (3.13) that the orientation factor for radial propagation (P, *SV*, Rayleigh) is $\cos^{2}\psi$ and that for tangential propagation (*SH*, Love) is sin⁡ψcos⁡ψ in terms of the angle ψ at which a plane wave component crosses the cable. If we multiply each response by tan⁡ψ we convert the radial term to sin⁡ψcos⁡ψ, i.e. that seen previously for the tangential, and convert the tangential term to $\sin^{2}\psi$ which no longer has the sign change that leads to stack cancellation. Since tan⁡ψ has a singularity when cos⁡ψ=0 at $\psi =90^{\circ }$, it is necessary to regularize such tangent multiplication in applying a modified stack. This can be done with exclusion of the immediate neighbourhood of the singularity where a zero factor is applied. The resulting modified stacks are shown in figure 7*e*,*f* for the same configuration as before. The modified radial stack (figure 7*e*: DAS-RL) is visually identical to the prior tangential stack in figure 7*d*, though the remapping has some very slight effects from the regularization. The modified tangential stack (figure 7*e*: DAS-TL) achieves the goal of stacking up Love waves at the desired slowness but the side bands are magnified by the slowness mapping in the DAS response.

The success of this angular weighting to enhance *SH* waves suggests that the application of an inverse slowness weight in stacking DAS records could help to improve the focusing of the array beam onto the correct slowness for body waves. To avoid the singularity at vertical incidence p=0, a regularization such as a water level would need to be applied to the inverse slowness.

Hence, if there is good control on the characteristics of arriving surface waves in terms of azimuth and approximate slowness, it is possible to extract Rayleigh waves by direct stacking of a DAS array with suitable geometry, and Love waves with an azimuth weighted stack using a regularized tangent weight. Stacking effects on linear cable runs will depend on their orientation relative to the arriving wavefronts.

Van den Ende & Ampuero [20] have made an empirical analysis of array beamforming for the March 2016 ML 4.3 Hawthorne earthquake recorded on both dense nodal seismometer and a complex geometry DAS array at the Brady Hot Springs site in Nevada. The event was about 150 km south of the site. Vertical component seismometer traces show strong coherence, but coherence is more patchy and geographically variable on the horizontal components. On the DAS array individual segments can show strong coherence, but the full suite of traces shows much less coherence than the seismometer results in part because of the influence of rapid changes in cable orientation across the Brady site and variable ground conditions [32]. Direct DAS stacking showed strong effects from local scattering magnified by the slowness weighting for slow arrivals, whereas conversion to ground velocity produced results comparable to those from the nodal array.

Steerable arrays require sites where good signal coherency is achieved to enhance signal strength. With the orientation effects of DAS recording, large numbers of abrupt changes of cable direction are undesirable since they complicate the array response. Complex sites such as the Brady geothermal field are good candidates for using the dense recording capabilities of DAS, but by their nature tend to distort the local strain wavefield, because of high sensitivity to small-scale heterogeneity [45]. Thus, not all DAS layouts can be expected to be suitable for array analysis, and a larger size than most current configurations is needed to get well focused beams.

## 6. Conclusion

Understanding of the nature of the seismic wavefield as recorded on a DAS cable requires recognition of the effect of local spatial averaging from the intrinsic gauge length and the important role of the orientation of cable segments relative to the seismic waves arriving at the site. Analysis in the slowness–frequency domain provides a convenient framework for including such effects.

At close ranges the influence of gauge length is strong and can be included by differencing ground velocity resolved along the fibre at the ends of the gauge interval. For more distant events, at seismic frequencies, the gauge averaging of strain rate is equivalent to extracting the acceleration resolved along the cable modulated by the horizontal slowness along the cable. Once the configuration of the cable has been determined, with calibration of DAS channels against physical position, it is possible to directly simulate the DAS signal using numerical integration of the frequency–slowness response. For teleseisms an effective approach is to employ a bundle of slowness around the geometrical arrival so that depth phase effects are correctly represented.

Not only does such synthesis allow an analysis of the different phase contribution to the observed wavefield with allowance for cable orientation, it also has a valuable role in allowing assessment of coupling effects along a cable. The relative simplicity of far-field results means that DAS recordings can be used in waveform inversion for source characteristics, alongside seismometer records.

The slowness domain representation of DAS recordings also provides a convenient way of examining the behaviour of different configurations of DAS cables. In particular, a single DAS layout can be employed as a steerable array for high-frequency waves. However, there is a tendency for the estimated slownesses for body waves to be slightly enlarged by the slowness component of the DAS response. Such effects are less important for surface waves, and by suitable weighting both Rayleigh and Love waves can be extracted from DAS stacks.

In this paper, I have concentrated on the use of horizontal DAS fibre configurations, and have demonstrated the way in which the orientation of the waves relative to the fibre has a strong control on the response. At a number of sites, cables have been deployed in vertical boreholes, and such recordings can have significant value for analysing many different types of seismic wave propagation phenomena in a lower noise environment (e.g. [46]). The analysis in terms of the slowness–frequency domain can be extended to downhole studies by modification of modelling codes for VSP simulation with differencing in the vertical direction, and orientation factors relative to the vertical rather than the horizontal.

## Data accessibility

The data used in the observational examples were provided for illustration of the nature of the DAS wavefield by the groups acknowledged below. The DAS modelling and array response codes are available from the AusPass repository (https://auspass.edu.au/research/DAS).
